# A case of lateral medullary infarction with severe dysphagia and worsening of respiratory failure in the chronic phase of recovery

**DOI:** 10.1002/ccr3.1641

**Published:** 2018-07-02

**Authors:** Yusuke Mon, Chisato Tamaki

**Affiliations:** ^1^ Department of Internal Medicine Kyoto Kyoritsu Hospital Ayabe Japan

**Keywords:** chronic phase of recovery, dysphagia, lateral medullary infarction, respiratory failure

## Abstract

Patients with lateral medullary infarction (LMI) sometimes present with respiratory failure in the acute or subacute phase. We experienced a LMI patient with progression of respiratory failure that required bilevel positive airway pressure in the chronic phase. LMI patients must be carefully observed even in the chronic phase of recovery.

## 1 INTRODUCTION

We experienced a patient with lateral medullary infarction (LMI) who showed progression of respiratory failure and required bilevel positive airway pressure. To the best of our knowledge, this is the first report of a LMI patient with progression of respiratory failure in the chronic phase of recovery.

Lateral medullary infarction (LMI) is caused by vertebral or posterior inferior cerebellar artery disease.^1^ LMI can be life‐threatening in the acute phase^2^; however, functional recovery is usually good.^3^

Here, we report on a patient with LMI who had severe dysphagia and chronic respiratory failure. To the best of our knowledge, this is the first report of a LMI patient with progression of respiratory failure that required bilevel positive airway pressure^4^ (bilevel PAP) in the chronic phase of recovery.

## 2 CASE REPORT

A 62‐year‐old man lost consciousness and was admitted to an emergency hospital. He was comatose (Hunt and Kosnik grade) and diagnosed with subarachnoid hemorrhage (SAH) due to rupture of a right vertebral artery dissecting aneurysm. Artery occlusion was performed, followed by ventricular drainage due to hydrocephalus. Nutrition was administered via a nasogastric tube. He gradually recovered consciousness but required ventilation for 1 month. After he was weaned from the ventilator, a speech cannula was installed at the tracheotomy site. The patient developed pneumonia and was treated with antibiotics. Two‐and‐a‐half months after SAH onset, he was transferred to our hospital for rehabilitation.

He had a history of hypertension and hyperlipidemia; however, he did not have a history of respiratory diseases or sleep disturbance. He did not smoke. The Epworth sleepiness scale (ESS)^5^ score was 7 before SAH.

On physical examination, the patient was 180 centimeters tall and weighed 82.6 kg with blood pressure 126/81 mm Hg, pulse 76/min, and respiratory rate 19 breaths/min with an O2 saturation (Spo2) of 88% on room air and 97% on 1 L of oxygen per minute. Cardiovascular examination was normal, the lungs were clear on auscultation, and abdominal examination was unremarkable. On neurological examination, he was alert with a MMSE score of 28. Eye movements were normal. There was mild narrowing of right palpebral fissure and a constricted pupil with preserved light reflex of the right eye. There was mild arm and hand paresis and moderate leg paresis on the right side. Deep tendon reflexes were brisk on the right side. The Babinski reflex on the right side was extensor. The right arm was ataxic, but the presence of ataxia was unequivocal in the right leg due to moderate paresis. Superficial sensation on the left side, including the face, was decreased, and deep sensation was preserved. The patient could not sit up or maintain a sitting posture and had bladder and bowel dysfunction.

Initial investigations revealed slight anemia (hemoglobin: 12.5 g/dL, mean cell volume: 94 μm^3^), hypoalbuminemia (3.5 g/dL), and low‐density to high‐density lipoprotein (HDL) cholesterol (26 mg/dL). Blood gas analysis revealed hypoxemia and hypercapnia (PaO2: 49.5 mm Hg, PaCO2: 73.4 mm Hg on room air and PaO2: 111.0 mm Hg, PaCO2: 77.7 mm Hg on 1 L of oxygen per minute). Other blood tests, including inflammatory markers (C reactive protein: 0.3 mg/dL), were normal. Respiratory function test results were vital capacity (VC) 3,022 mL, %VC 73%, forced expiratory volume in 1‐second (FEV_1.0_) 2,540 mL, and FEV_1.0%_ 78%.

**Figure 1:**
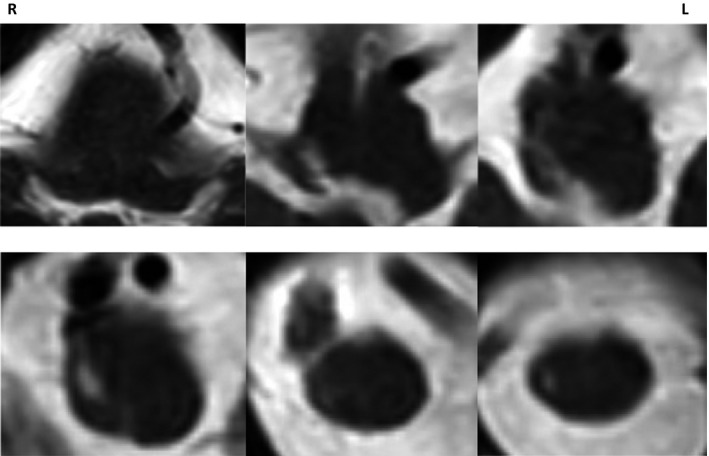
Magnetic resonance T2‐weighted image (MRI‐T2WI) of the medulla oblongata and the upper cervical cord

Head magnetic resonance imaging (T2‐weighted MRI) revealed a focal high‐intensity area in the dorsolateral medulla oblongata and the upper cervical cord on the right side (Figure 1).

Thoracic computed tomography (CT) was normal.

Neurological examination and MRI showed lateral medullary syndrome with ipsilateral palsy.

Through physical and occupational therapy, he gradually developed his physical strength. The right leg paresis recovered from a moderate to slight degree of impairment, and the presence of ataxia became apparent.

We evaluated his swallowing ability by videofluorography and videoendoscopy. Videofluorography showed that relaxation of the upper esophageal sphincter (UES), especially on the right (ipsilateral) side, was insufficient. Barium jellies hardly passed the UES with passage of small amounts along the left (contralateral) side, and after several swallowing actions, aspiration into the trachea occurred. Videoendoscopy showed insufficient movement of the right vocal cord. We conducted dysphagia rehabilitation. However, improvement was limited, and gastrostomy tube was installed. He received combined cricopharyngeal myotomy and laryngeal elevation 1 year and 7 months after SAH onset. After surgery, swallowing ability improved. Finally, he could take almost all nutrition orally in an upright sitting position.

**Figure 2:**
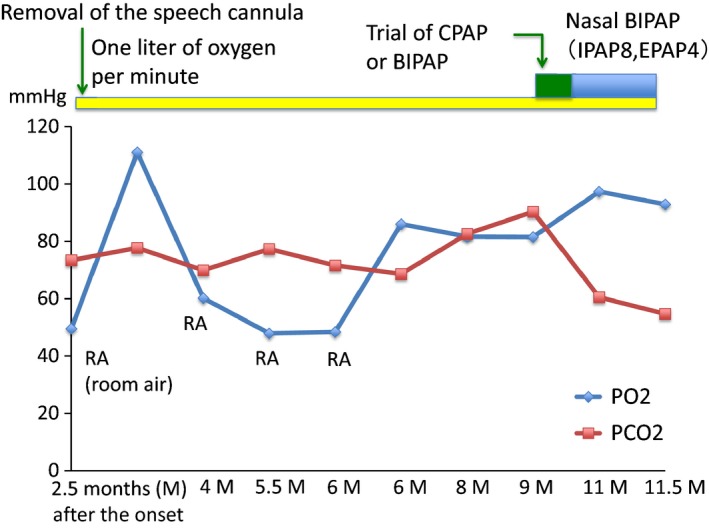
Respiratory condition during the course

We removed the speech cannula soon after admission. We observed his respiratory condition, and it was expected to improve. However, blood gas analysis did not ameliorate and PaCO2 began to worsen. Nine months after SAH onset, PaCO2 became 90.3 mm Hg without the presence of respiratory infection. Portable polysomnography (PSG) showed that consecutive apnea‐hypopnea indices (AHIs) were 48.6 and 63.0/h. He was diagnosed with sleep apnea syndrome (SAS). Continuous positive airway pressure (CPAP) or bilevel positive airway pressure (bilevel PAP) was tried under various conditions, and an appropriate condition was eventually found. He received nasal bilevel PAP under the condition of spontaneous timed (ST) mode (IPAP of 8 cm H2O, EPAP of 4 cm H2O, and backup breathing frequency of 12/min) during the night. His nocturnal SpO2 dramatically improved under this condition. Blood gas analysis during the daytime also improved (PaO2: 92.9 mm Hg and PaCO2: 54.7 mm Hg on 1 L of oxygen per minute) (Figure 2).

He was eventually discharged home 2 years and 2 months after SAH onset. Then, he needed no oxygen supply (PaO2: 75.4 mm Hg and PaCO2: 50.6 mm Hg on room air). His weight decreased to 74.9 kg at the time of discharge. However, it recovered to 80.3 kg 9 months after discharge. His neurologic manifestation remained unchanged.

## 3 DISCUSSION

This patient showed lateral medullary syndrome with ipsilateral palsy, which is called Opalski syndrome.^6^ This syndrome is rare, and the functional recovery is not fully understood. Nelles et al^3^ reported that patients with LMI have few functional deficits after completion of inpatient rehabilitation, continue to improve functionally after discharge, and often resume their previous activities. However, it is sometimes difficult to rehabilitate patients with LMI, especially those with dysphagia. We conducted dysphagia rehabilitation, and it was useful to some extent. However, this patient needed surgery to take almost all nutrition orally.

Respiratory failure was another difficult problem in this patient. Breathing depends on a central pattern generator that includes neurons located in the dorsolateral pons, nucleus of the solitary tract (NTS), and ventrolateral medulla (ventral respiratory column).^7^ Disturbance of automatic and voluntary breathing is observed in these lesions.^8^ Portable PSG is useful for the diagnosis of SAS.^9^ Our portable PSG cannot detect respiratory effort; therefore, it was difficult to distinguish between obstructive and central SAS. However, this patient had no respiratory diseases or sleep disturbance. The ESS score before the onset of disease was low and SAS started after the onset of LMI. Therefore, we considered a diagnosis of central SAS and the worsening of PaCO2 even in the daytime indicated disturbance of both automatic and voluntary breathing. CPAP is effective for treating obstructive SAS but not alveolar hypoventilation.^10^ Noninvasive ventilation is recommended in alveolar hypoventilation.^10^ Therefore, we introduced bilevel PAP as noninvasive ventilation and his respiratory condition improved.

Norrving et al^2^ reported that 5 patients of 43 (11.6%) died from respiratory and cardiovascular complications in the acute phase of LMI. There are many reports of LMI requiring respiratory assistance, mostly in the acute phase. Several patients have been reported to require respiratory assistance in the subacute phase ranging from 7 days to 3 months.^8^, ^11^, ^12^, ^13^, ^14^, ^15^ However, to the best of our knowledge, this is the first report of a patient who showed progression of respiratory failure and required bilevel PAP in the chronic phase of recovery. Mendoza et al^8^ speculated that secondary neuronal degeneration, apoptosis, or abnormal plasticity involving local synaptic interconnections may explain the delay in the development of respiratory failure after lateral medullary infarction, ranging several days to several months. Their explanation may be applicable to this patient.

Several neurological diseases such as amyotrophic lateral sclerosis, myasthenia gravis, and myopathy, or malignant diseases lead to respiratory failure. However, his neurologic manifestation remained unchanged for more than 2 years. His weight decreased 7.7 kg at the time of discharge but recovered 9 months after discharge. Therefore, it is unlikely that he has another neurological disease or malignant disease. We believe that his respiratory failure is due to LMI.

This patient did not face catastrophic respiratory failure and used preventive respiratory assistance through bilevel PAP. Although the mechanism by which respiratory failure worsens in the chronic phase is unclear, we emphasize that patients with LMI must be carefully observed even in the chronic phase of recovery.

## CONFLICT OF INTEREST

None declared.

## AUTHORSHIP

YM: wrote and elaborated the manuscript, and CT: revised the manuscript.
